# Bacterial and Archaeal Diversity and Abundance in Shallow Subsurface Clay Sediments at Jianghan Plain, China

**DOI:** 10.3389/fmicb.2020.572560

**Published:** 2020-10-22

**Authors:** Dandan Song, Zhou Jiang, Teng Ma, Yiran Dong, Liang Shi

**Affiliations:** ^1^School of Environmental Studies, China University of Geosciences, Wuhan, China; ^2^State Key Laboratory of Biogeology and Environmental Geology, China University of Geosciences, Wuhan, China

**Keywords:** bacteria, archaea, diversity and abundance, clay sediment, Jianghan Plain

## Abstract

Clay layers are common in subsurface where microbial activities play an important role in impacting the biogeochemical properties of adjacent aquifers. In this study, we analyzed the community structure and abundance of bacteria and archaea in response to geochemical properties of six clay sediments at different depths in a borehole (112°34′0″E, 30°36′21″N) of Jianghan Plain (JHP), China. Our results suggested that the top two clay layers were oxic, while the remaining bottom four clay layers were anoxic. Both high-throughput sequencing and qPCR of 16S rRNA gene showed relatively high abundance of archaea (up to 60%) in three of the anoxic clay layers. Furthermore, microbial communities in these clay sediments showed distinct vertical stratification, which may be impacted by changes in concentrations of sulfate, HCl-extractable Fe^2+^ and total organic carbon (TOC) in the sediments. In the upper two oxic clay layers, identification of phyla Thaumarchaeota (11.2%) and Nitrosporales (1.2%) implied nitrification in these layers. In the two anoxic clay layers beneath the oxic zone, high abundances of *Anaeromyxobacter*, Chloroflexi bacterium RBG 16_58_14 and Deltaproteobacteria, suggested the reductions of nitrate, iron and sulfate. Remarkably, a significant portion of Bathyarchaeota (∼25%) inhabited in the bottom two anoxic clay layers, which may indicate archaeal anaerobic degradation of TOC by these organisms. The results of this study provide the first systematic understandings of microbial activities in subsurface clay layers at JHP, which may help develop microorganism-based solutions for mitigating subsurface contaminations.

## Introduction

Clays and clay minerals are abundant in soils, sediments, and subsurface (Dong et al., 2009; Dong, 2012). In subsurface, clays and clay minerals are often found in the sediment layers with different depths, where they impact microbial activities substantially. For example, iron (Fe)-rich clays and clay minerals function as electron donors and/or acceptors to support microbial growth (for reviews, see Dong et al., 2009; Dong, 2012; Shi et al., 2016). The clays rich in aluminum oxides (Al_2_O_3_), however, inhibit the growth of sulfate-reducing bacteria (SRB) (Wong et al., 2004). This is consistent with the observations that microbial sulfate reduction activity in subsurface clay layers is lower compared to that in the adjacent sand layers (McMahon and Chapelle, 1991; Ulrich et al., 1998). It was also suggested that microorganisms in the subsurface clay layers converted complex organic matters into low-molecular-mass fatty acids and hydrogen gas (H_2_) via fermentation. These fermentation products diffused into the surrounding environments (e.g., aquifers) where they were used by SRB as electron donors to reduce sulfate (McMahon and Chapelle, 1991; McMahon et al., 1992; Krumholz et al., 1997; Fredrickson and Balkwill, 2006). Furthermore, recent results demonstrated that dissolved organic carbon diffused from the adjacent clay layer activated the microbial activity in aquifers, which increased the arsenic (As) level in the groundwater of aquifers (Mihajlov et al., 2020). Thus, microorganisms in the subsurface clay layers impact the geochemical properties of not only the clay layers, but also their surrounding environment, such as the groundwater of aquifers. Finally, microbial activity in subsurface clay layers also influences the diagenesis of subsurface sediments, such as aquitard (McMahon et al., 1992; Hesse and Schacht, 2011).

In addition to Fe(III)-reducing, Fe(II)-oxidizing and fermentation microorganisms, other microorganisms have been identified from subsurface clay layers. Bacteria related to Proteobacteria and Firmicutes were found in a clay layer that was 224 m deep (Boivin-Jahns et al., 1996). Analyses of different clay layers of coastal subseafloor sediments from Okhotsk sea identified a variety of bacteria and archaea, such as green non-sulfur bacteria and deep sea archaeal group (Inagaki et al., 2003). Different groups of bacteria were found in the Opalinus clay rock from Mont Terri, Switzerland and Kisameet Glacial clay from British Columbia, Canada, whose functions range from CO_2_ fixation to sulfate reduction (Bagnoud et al., 2016a, b; Svensson et al., 2017). Thus, a phylogenetically and physiologically diverse groups of bacteria and archaea are associated with subsurface clay layers.

Jianghan Plain (JHP) is a semi-closed Quaternary basin in Hubei province, China, which was formed by the alluvial sediments of the Yangtze River and Han River (Gan et al., 2014). Because of intensive human activities, the aquifer systems of JHP has been polluted with different contaminants, such as ammonium (Du et al., 2017), As (Gan et al., 2014), antibiotics (Tong et al., 2014), and phthalate esters (Liu et al., 2010), which pose substantial health risks to the local residents. Thus, the JHP subsurface has been subjected to rigorous investigation in order to find the science-based solutions for mitigating the risks. For example, microbiological investigation of the core samples from the shallow subsurface of JHP revealed that the subsurface bacterial communities and functional groups were influenced by a variety of environmental factors, such as pH, Fe content, and NH_4_^+^. Results also showed existence of unique dissimilatory arsenate-reducing bacterial communities in the JHP sediments (Lu et al., 2017). Further analyses suggested that Fe(III)-reducing and Fe(II)-oxidizing bacteria play crucial roles in controlling As dynamic in the shallow sediments of JHP (Deng et al., 2018; Zheng et al., 2019). Moreover, up to 192 bacteria species with pathogenic potential were identified from 156 samples collected from JHP groundwater (Wu et al., 2019). Geochemical analyses also revealed that the shallow aquifer system (<50 m below ground surface) in JHP consisted of multiple layers rich in clays (Deng et al., 2018; Gan et al., 2018). However, the bacterial and archaeal communities in these clay layers had never been systematically investigated.

In this study, we used high-throughput sequencing and q-PCR of 16S rRNA gene to investigate the bacterial and archaeal community structure and abundance of six clay sediments at different depths in a borehole from JHP shallow subsurface. We further analyzed vertical distribution characteristics of microbial community structure and functional potentials and determined environmental factors that significantly impact the microbial community structure.

## Materials and Methods

### Site Description and Sampling

**FIGURE 1:**
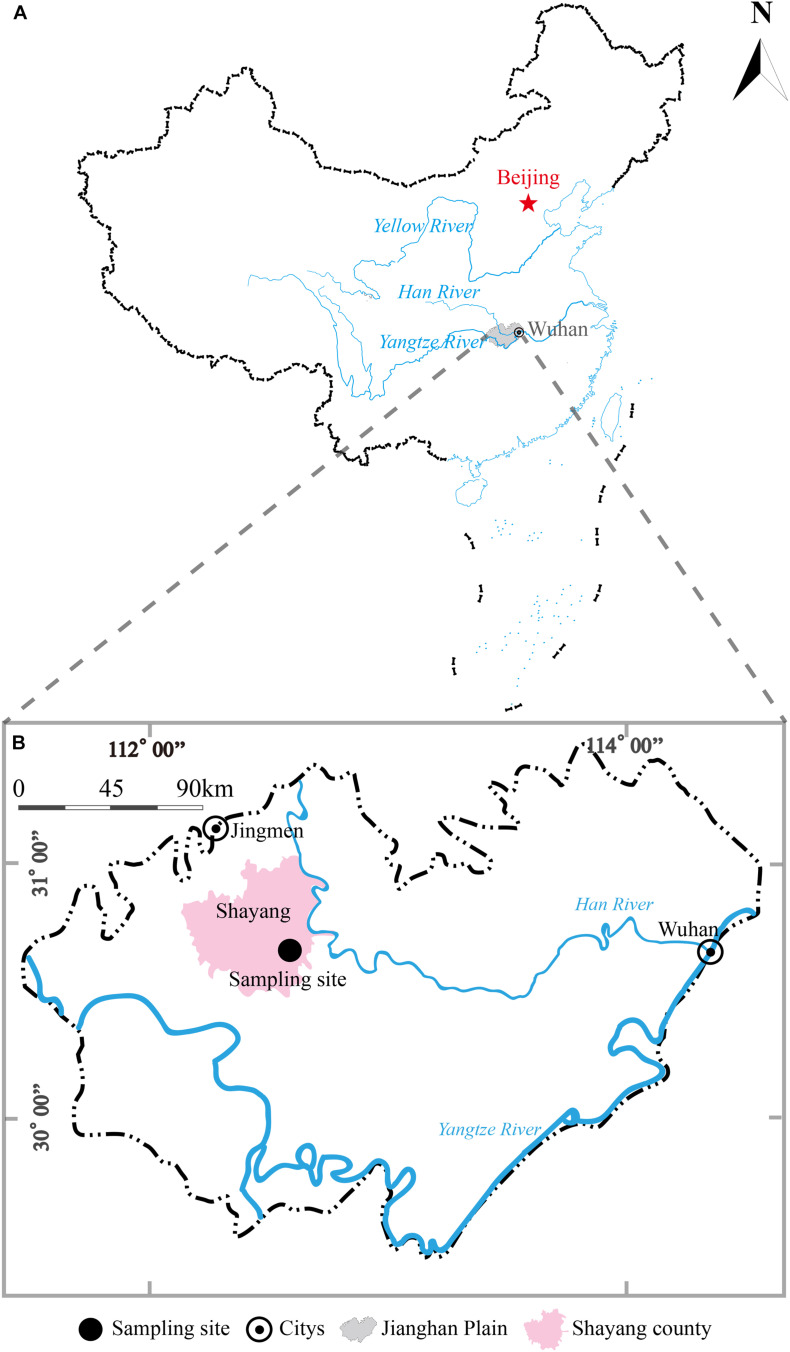
A geographic map showing locations of Jianghan Plain, China **(A)** and borehole site for clay sediment sampling in Jianghan Plain **(B)**. The shadow area in map **(A)** refers to the boundary of Jianghan Plain.

The sampling site (112°34′0″E, 30°36′21″N) was near a paddy field at Guangmang village, Shayang county, Jingmen city, Hubei province, China (Figure 1). This sampling site is located in the northern part of JHP and lies on the western bank of Hang River. A borehole with a depth of 30 m was drilled in September, 2017 using direct-mud rotary drilling technique. Based on the lithological characters in this borehole, six representative clay sediments samples were collected from the depths of 3, 5, 7, 9, 12, and 26 m and were placed in sterile anaerobic bag (Lu et al., 2017). All the samples were stored and transported on dry ice in the field. The samples along the depth were herein designated A1, A2, A3, A4, A5, and A6, respectively.

### Geochemical Analyses

In the laboratory, the external portions of the sediment cores that may be contaminated and oxidized during sampling were carefully removed in an anaerobic chamber filled with 97% N_2_ and 3% H_2_ (Coy Laboratory Products, MI, United States) (Lu et al., 2017). Only the internal parts were used for further analyses. Each sediment sample was homogenized and split into two portions to perform geochemical measurements and DNA extraction, respectively. Prior to chemical analysis, sediment samples were freeze-dried, homogenized and filtered through a 2.0 mm sieve. The pH value was analyzed using a Delta 320 pH Analyzer (Mettler Toledo, OH, United States) in a suspension of 5 g sediment and 25 mL 0.01 M calcium chloride. Concentrations of anion were measured by ion chromatograph (Dionex Aquion, Thermo Fisher Scientific, MA, United States) equipped with an ion exchange column (Dionex IonPac AS22 IC, Thermo Fisher Scientific, MA, United States) after extraction with ddH_2_O (a sediment-to-water ratio of 1:3). HCl-extractable Fe^2+^ was determined using the Ferrozine-based assay (Islam et al., 2004). Total Fe (Fe_Tot_), Mn (Mn_Tot_), and As (As_Tot_) concentrations in the sediments were extracted by aqua regia and measured by inductively coupled plasma optical emission spectrometer (ICP-OES, Avio 200, PerkinElmer, MA, United States) and atomic fluorescence spectrometry (AFS-9780, Haiguang, Beijing, China), respectively. Total organic carbon (TOC) was determined by a TOC analyzer (Vario TOC select, Elementar, Hesse, Germany). All the samples were run in triplicates.

### DNA Extraction, 16S rRNA Gene Amplification and High-Throughput Sequencing

Genomic DNA in samples were extracted using DNeasy PowerMax Soil Kit (Qiagen, Shanghai, China) following the manufacturer’s instructions. DNA concentrations and quality were determined by a Nanodrop One UV-Vis Spectrophotometer (Thermo Fisher Scientific, MA, United States). The V4 region of bacteria and archaeal 16S rRNA gene was amplified using the primer 515F (5′-GTGCCAGCMGCCGCGGTAA-3′) and 806R (5′-GGACTACHVGGGTWTCTAAT-3′) combined with Illumina adapter sequences, a pad and a linker, as well as barcodes on the reverse primers (Jiang et al., 2016). PCR amplification was carried out in a 50 μL reaction buffer containing 25 μL Premix *Ex Taq* (TaKaRa Biotechnology, Dalian, China), 0.5 μM of both forward and reverse primers, 60 ng DNA template using the following program: 5 min at 94°C for initialization; 30 cycles of 30 s denaturation at 94°C, 30 s annealing at 52°C, and 30 s extension at 72°C; 10 min final elongation at 72°C. Triplicate PCR reactions were performed per sample and pooled together. After confirmed by agarose gel electrophoresis, PCR products were mixed in equidensity ratios according to the GeneTools Analysis Software (Version 4.03.05.0, SynGene, Karnataka, India) and then purified with EZNA Gel Extraction Kit (Omega Bio-tek, GA, United States) (Chen et al., 2020). Sequencing libraries were prepared using NEBNext Ultra DNA Library Prep Kit for Illumina (New England Biolabs, MA, United States) following the manufacturer’s recommendations. The library was sequenced on an Illumina Hiseq 2500 platform (Guangdong Magigene Biotechnology, Guangzhou, China) and 250 bp paired-end reads were generated. Raw sequencing read of 16S rRNA gene in the study were deposited in the NCBI Sequence Read Archive under the accession number SRP216165.

### Sequencing Data Processing and Statistical Analyses

Raw sequence read of 16S rRNA with perfect matches to barcodes were split to sample libraries. Forward and reverse reads with at least 30 bp overlap and less than 25% mismatches were joined using Fast Length Adjustment of SHort reads (FLASH) (Magoc and Salzberg, 2011). The joined sequences were trimmed using Btrim with a QC threshold of greater than 30 over a 5 bp window size and a minimum length of 150 bp (Kong, 2011). After trimming the sequences containing N base and sequences with the lengths of <248 or >255 bp, the clean sequences were clustered to operational taxonomic units (OTUs) by Uparse at a similarity level of 97% (Edgar, 2013). For each representative sequence in OTUs, the silva^1^ database (SSU r132) was used to annotate taxonomic information at a minimal 50% confidence. Singletons in generated OTU tables were removed and then samples were rarefied at 42,887 sequences per sample based on the least number of sequences in the samples.

All statistical analyses in the study were performed with the Vegan and Stats packages in R^2^ (Jiang et al., 2016). Alpha diversity indices, such as Chao1, Shannon and equitability, and library coverage were calculated. Correlation analysis was used to evaluate the relationship between geochemical variables and diversity indices as well as microbial community compositions. The differences of microbial community composition among samples were determined using principal coordinates analysis (PCoA) (Wang et al., 2016). Three complimentary non-parametric multivariate statistical tests including adonis, ANOISM, and MRPP was performed to test the significance of microbial community differences among different groups (Jiang et al., 2016). Canonical correspondence analysis (CCA) was performed to elucidate the relationship between microbial community composition and geochemical variables. Geochemical variables were chosen for CCA analysis based on their significance calculated from individual CCA results and variance inflation factors (VIFs) calculated during CCA (Van Nostrand et al., 2009). Partial CCA (pCCA) was used to partition variation observed in microbial community composition to these selected geochemical variables (Lu et al., 2012b).

### Quantitative Polymerase Chain Reaction

The universal primers bac331F (5′-TCCTACGGGAGGCAGC AGT-3′)-bac797R (5′-GGACTACCA GGGTCTAATCCT GTT-3′) and arch349F (5′-GYGCASCAGKCGMGAAW-3′)- arch 806R (5′-GGACTACVSGGGTATCTAAT-3′) were employed for quantification of bacterial and archaeal 16S rRNA gene, respectively. The bacterial 16S rRNA gene from *Shewanella surugensis* strain c959 and archaeal 16S rRNA gene from *Methanospirillum* sp. clone sagar113 were synthesized (Lin et al., 2012b) and cloned into pUC57-Kan as the quantitative polymerase chain reaction (qPCR) standards. PCR amplification was carried out in a 20 μL reaction buffer containing10 μL PowerUp SYBR Green Master Mix (Applied Biosystems, Thermo Fisher Scientific, MA, United States), 0.5 μM of both forward and reverse primers, 60 ng DNA template using the following program: 2 min at 95°C for initial denaturation; 40 cycles of 15 s denaturation at 95°C, 60 s annealing/extension at 60°C. All reactions were performed in triplicates. The melting curve analysis was performed to verify reaction specificity. The correlation coefficients (R^2^) of linear plots between the Ct value and log (copy numbers/reaction) for bacterial and archaeal 16S rRNA genes were 0.995 and 0.992, respectively. The qPCR amplification efficiencies were in the range of 92–103%.

## Results and Discussion

### Sediment Geochemistry

**FIGURE 2:**
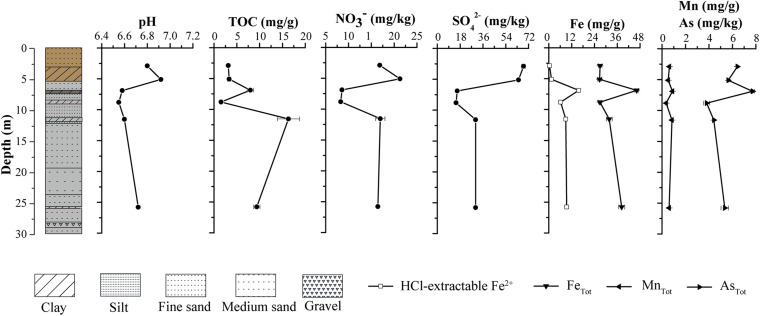
Vertical lithological profile of the borehole (left part) and physicochemical properties of sediment samples at different depths from the borehole (right part). The lithologies in the borehole were represented with the colors and textures defined for sediments at corresponding depths. The boxes represent core textures, which include clay, silt, fine sand, medium sand, and gravel. The colors depict the physical appearance of the core. The top portion of the core that includes A1 and A2 clay layer appears yellow-brown, while the remaining portion appears gray.

The grain size of sediments along the borehole generally ranged from clay to silt to fine-medium sand. The sediment color shifted dramatically from yellow-brown in upper layer (0–6 m) to gray in lower layer (6–30 m) (Figure 2). The physical properties of sediments in the borehole implied that an interface of vadose and saturated zone may occur near 6 m below the ground, which is very close to water level in JHP reported by Gan et al. (2018). Distinct geochemical characteristics occurred among upper layer samples (A1 and A2), middle layer samples (A3 and A4) and bottom layer samples (A5 and A6) (Figure 2). Sediment pH values were slightly acid, ranging from 6.55 at sample A4 to 6.92 at sample A2 (Figure 2). Nitrate and sulfate concentrations displayed a similar vertical distribution. The highest levels of nitrate and sulfate were found at sample A1 and A2, while the lowest were at sample A3 and A4 (Figure 2). TOC contents ranged from 1.54 mg/g (sample A4) to 16.31 mg/g (sample A5). High levels of Fe, Mn, and As were detected in all the samples, which is comparable to those reported before (Gan et al., 2014). The sample A3 contained the highest level of Fe, Mn, and As, while the A4 contained the lowest. Samples A1 and A2 contained few HCl-extractable Fe^2+^, which was detected in all other samples and their levels were much lower than that of total Fe detected. Similar to that for total Fe and Mn, the highest level of HCl-extractable Fe^2+^ was detected in sample A3 (Figure 2). Compared to that in samples A1 and A2, sample A3 contained much lower levels of nitrate and sulfate and higher level of Fe^2+^. These results suggest a change from microbial aerobic respiration of O_2_ in samples A1 and A2 to anaerobic respiration of nitrate, sulfate and Fe^2+^ at sample A3 as well as an oxic to anoxic transition zone between the depths of samples A2 and A3.

### Alpha Diversity of Microbial Community and Abundance of Bacteria and Archaea

**FIGURE 3:**
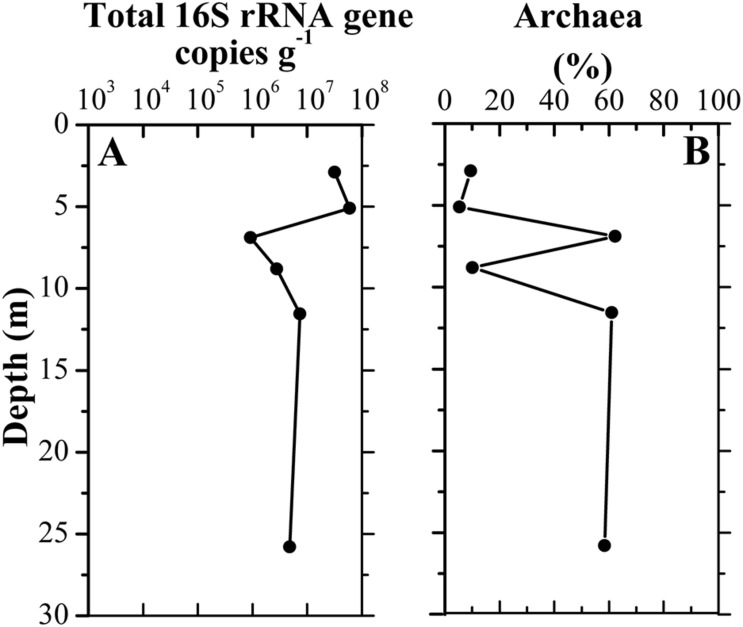
Vertical distribution of total 16S rRNA gene copy numbers **(A)** in clay sediments with different depths and the relative abundance of archaeal copy numbers to total 16S rRNA gene copy numbers **(B)**.

A total of 328,827 sequences were originally obtained from six sediment samples. After rarefaction at 42,887 sequences per sample, 257,322 sequences remained. A large number of taxa were detected in those samples, with 1,990–3,848 observed OTUs and 2,492–4,250 predicted OTUs (Chao1) (Supplementary Table 1). Library coverage of those samples ranged from 78.95 to 91.02%. Alpha diversity indices including richness, Shannon diversity and equitability generally declined along the borehole (Supplementary Figure 1). Moreover, Shannon diversity displayed a significantly negative relationship with depth (*r* = −0.829, *p* = 0.042). qPCR results showed the abundance of total 16S rRNA gene in the samples ranged from 9.26 × 10^5^ to 5.96 × 10^7^ copies/g sediment. The samples A3–A6 had approximately 10-fold less than the samples A1–A2 in 16S rRNA gene copies (Figure 3). The concurrent decrease of alpha diversity indices and 16S rRNA gene abundance along the borehole are most likely attributed to change of redox condition. This is consistent with geochemical data that suggest an oxic to anoxic transition between the depths of sample A2 and A3. Previous results also demonstrated that compared to that in the oxic zone of a terrestrial subsurface, microbial population was much lower in the anoxic zone (Lin et al., 2012a). Remarkably, archaea in these sediments constituted a far larger proportion of the whole microbial community (35.5% on average), as compared to other borehole sediments in aquifer system (Lin et al., 2012a). In contrast to that (5.4–9.4%) in the samples A1, A2, and A4, the relative abundances of archaea in samples A3, A5, and A6 were up to ∼60%, which suggests a dominant role of archaea in regulating biogeochemical reactions in these subsurface clay layers (Castelle et al., 2015; Anantharaman et al., 2016). A significantly positive correlation between archaeal relative abundance and TOC content (*r* = 0.846, *p* = 0.034) implied that extremely low TOC content in sample A4 (Figure 2) might lead to dramatically drop of archaeal relative abundance.

### Microbial Community Composition and Function

**FIGURE 4:**
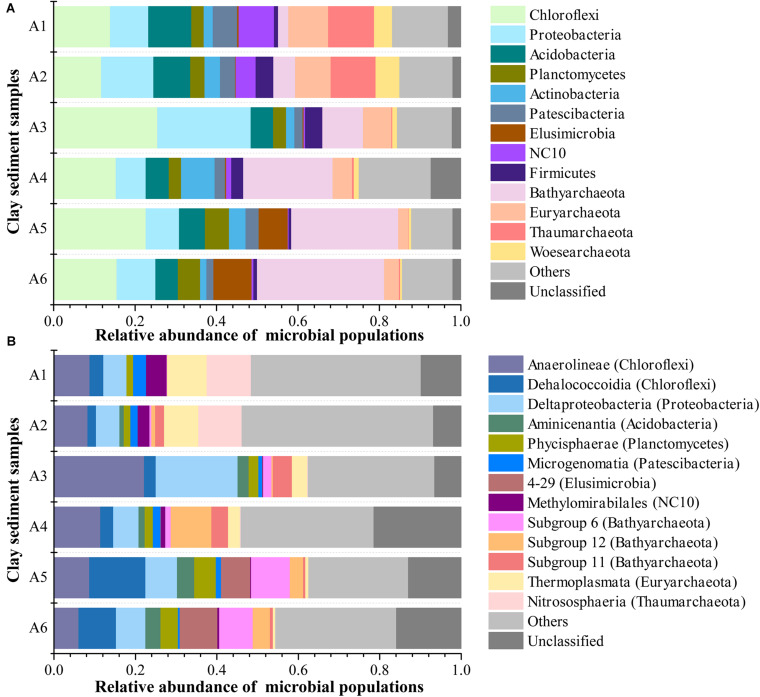
Microbial community structure of sediment samples at the **(A)** phylum- and **(B)** class-level. Only top 13 phyla and classes in abundance were displayed, the rest phyla and classes were summed and assigned to others.

**FIGURE 5:**
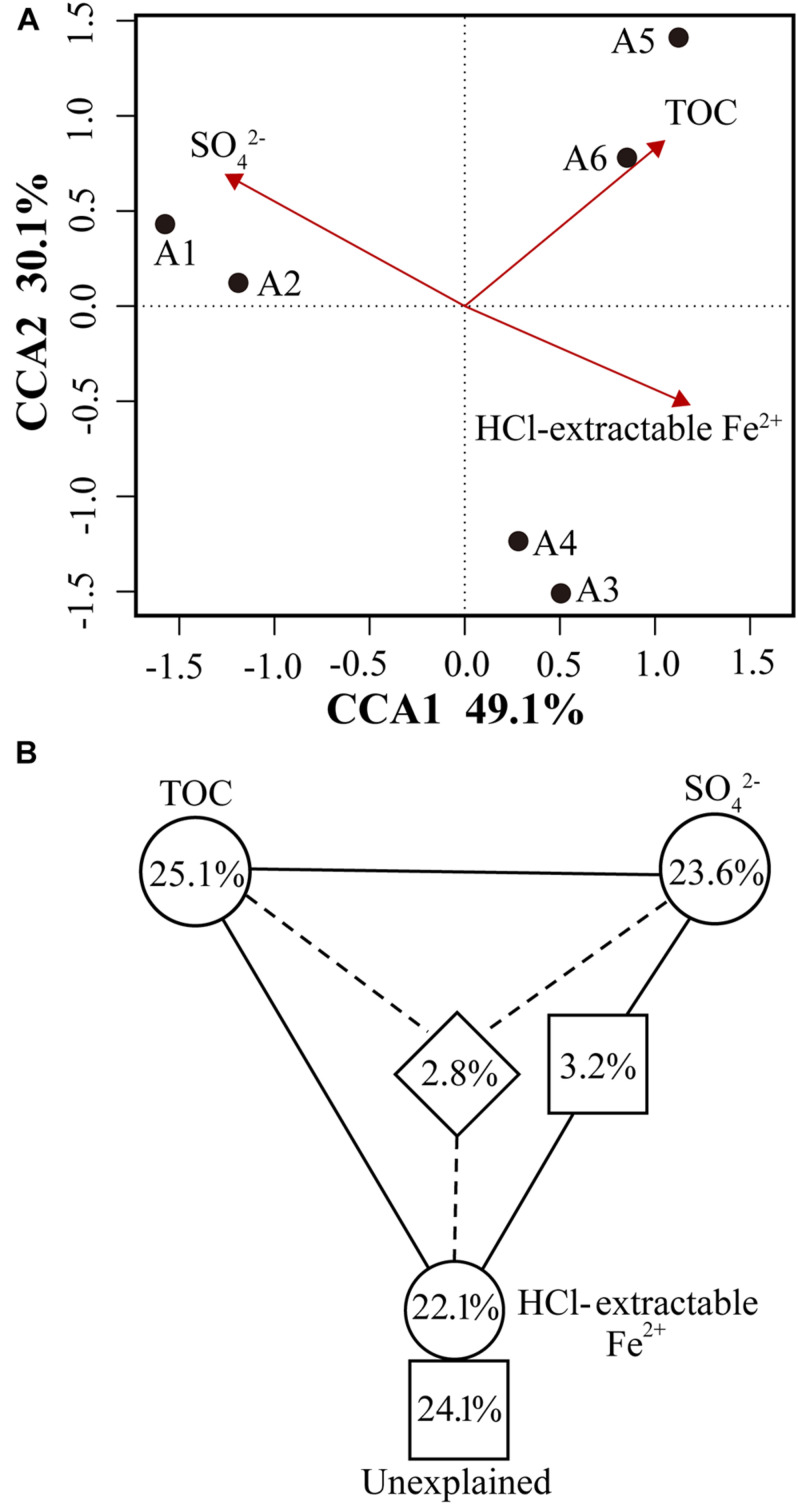
**(A)** Canonical correspondence analysis (CCA) of microbial community composition and geochemical variables (TOC, sulfate, HCl-extractable Fe^2+^). Geochemical variables were chosen based on significance calculated from individual CCA results and VIFs calculated during CCA. The percentage of variation explained by each axis is shown, and the relationship is significant (*p* = 0.014). **(B)** Variation partitioning of microbial community composition respond to significant geochemical variables.

The microbial community in all six clay sediments generally comprised 67.5% bacterial phyla, 29.5% archaeal phyla, and 3% unclassified phyla (Figure 4A). The dominant phyla in bacteria mainly included Chloroflexi (17.5%), Proteobacteria (11.7%), Acidobacteria (7.1%), Planctomycetes (4.0%), Actinobacteria (3.7%), Patescibacteria (3.2%), Elusimicrobia (2.9%), NC10 (2.6%), and Firmicutes (2.3%), with respect to Bathyarchaeota (16.2%), Euryarchaeota (6.1%), Thaumarchaeota (3.9%), and Woesearchaeota (2.3%) being abundant in archaea (Figure 4A). The presence of 3% unclassified phyla in this study implied that subsurface clay layers in JHP maybe also nourish many novel bacterial or archaeal organisms as same as the aquifer adjacent to the Colorado River, near Rifle, CO, United States (Castelle et al., 2015; Anantharaman et al., 2016), which is deserved for the further investigation. The relative abundance of archaea revealed by 16S rRNA gene sequencing was comparable to that from q-PCR results, which highlights the importance of archaea in those samples. Microbial community composition in JHP subsurface clay layers is very similar to the aquifer adjacent to the Colorado River, both of which have dominant Chloroflexi, Proteobacteria and some archaeal phyla (Castelle et al., 2013; Hug et al., 2013). Vertically, NC10, Euryarchaeota, Thaumarchaeota, and Woesearchaeota were mainly found in samples A1 and A2, whereas Bathyarchaeota and Elusimicrobia dominate in samples A5 and A6. Besides, the abundances of Bathyarchaeota and Elusimicrobia in clay sediments had a significantly positive correlation with depth (Supplementary Table 2). Chloroflexi and Proteobacteria were predominant in the sample A3. At the class level, microbial community composition was more diverse and even, and about 12.2% sequences were not classified to known classes, suggesting the presence of novel lineages in these samples (Figure 4B). Compared to the relatively high proportions of Nitrososphaeria, Thermoplasmata, and Methylomirabilales in the upper samples A1 and A2, the bottom sediments from A5 and A6 were characterized by the presence of Dehalococcoidia of Chloroflexi, 4–29 of Elusimicrobia, Subgroup 6 and 12 of Bathyarchaeota. In addition, sample A3 was dominated by Anaerolineae and Deltaproteobacteria as well as subgroup 11 of Bathyarchaeota. Based on all OTUs detected, both PCoA and CCA ordination analyses showed that the microbial communities were similar between samples A1 and A2, between samples A3 and A4, and between samples A5 and A6, respectively, whereas microbial communities among the three groups (A1/A2, A3/A4, and A5/A6) were far apart (Figure 5A and Supplementary Figure 2), suggesting a distinct difference of microbial community among these three different zones of clay layers. Furthermore, the difference of microbial community among upper layer samples (A1 and A2), middle layer samples (A3 and A4) and bottom layer samples (A5 and A6) was tested to be significant, revealed by Adonis analysis (Supplementary Table 3). All of these results demonstrated the unique microbial communities resided in different subsurface clay layers of JHP.

Consistent with geochemical characteristics in the sediment samples, the function potentials of microbial community also displayed a distinct distribution along the depth. In the clay sediments of A1 and A2, nitrification was identified. Ammonia-oxidizing archaea (AOA) might outcompete ammonia-oxidizing bacteria (AOB) and contribute to ammonia oxidation in upper clay sediments, as indicated by high abundance of Thaumarchaeota (Nitrosopumilales and Nitrososphaerales) relative to these representative AOB (Supplementary Figure 3; Herber et al., 2020). Nitrite produced by AOA could serve as substrate for nitrite-oxidizing bacteria (NOB), such as Nitrosporales, leading to nitrate accumulation in upper samples (Figure 2; Daims et al., 2016). AOA and NOB abundance significantly positively correlated to sulfate concentration and negatively correlated to HCl-extractable Fe^2+^ concentration, which further suggested the oxic condition in samples A1 and A2 (Supplementary Figure 2 and Supplementary Table 2). In addition, Woesearchaeota were mainly found in upper sediment samples from the borehole (Supplementary Figure 3), which coincide with its wide ecological niches ranging from oxic to anoxic biotopes and might provide a series of intermediates for other aerobes and anaerobes due to reported heterotrophic and syntrophic lifestyle (Castelle et al., 2015; Liu et al., 2018). The sediments of A3 and A4 were characterized by the significant portion of microbial populations associated with reductions of nitrate (*Anaeromyxobacter* and nitrate-dependent methanotrophic *Methanoperedens*-like archaea), Fe (*Anaeromyxobacter* and Chloroflexi bacterium RBG_16_58_14), and sulfate (Deltaproteobacteria) (Supplementary Figure 3; He and Sanford, 2003; Muyzer and Stams, 2008; Anantharaman et al., 2016; Welte et al., 2016; Bell et al., 2020). This is consistent with observations of low levels of nitrate and sulfate, but high level of HCl-extractable Fe^2+^ in the samples A3 and A4, which further support the idea of an oxic to anoxic transition between the sediments of A2 and A3 (Figure 2). These microbial groups may use TOC as energy and electron sources to reduce nitrate, Fe, and sulfate. More importantly, reduction of iron and sulfate causes As mobilization in JHP groundwater system (Deng et al., 2018; Zheng et al., 2019). In aquifers, As is mainly adsorbed to solid-phase iron minerals (Fendorf et al., 2010). Indeed, our results also showed that amounts of As_Tot_ detected correlated with those of Fe_Tot_ and HCl-extractable Fe^2+^, which are all detected in the highest amounts in sample A3 (Figure 2). These iron-reducing bacteria and sulfide produced by SRB in sample A3 most likely reductively dissolve the iron minerals adsorbed with As, which could release adsorbed As into the pore water of clay sediments. It has documented that the pore water containing dissolved As and As-mobilizing solutes, such as dissolved organic carbon and competing ions, are expelled to the underlying aquifer with extensive groundwater pumping, thus making As contamination in aquifers even worse (Erban et al., 2013; Mihajlov et al., 2020; Xiao et al., 2020). Methanogens, such as Methanobacteriales, Methanocellales, and Methanosarcinales, were also relatively abundant in A3 and A4 sediments, where methane might be oxidized by methanotrophic microorganisms by coupling with nitrate reduction (Supplementary Figure 3). Significantly different with A1–A4 sediments, a substantial portion of Bathyarchaeota (∼25%) consisting of mainly subgroup 6 and subgroup 12 were identified in the bottom A5 and A6 clay layers. Given the common acetyl-CoA-centralized heterotrophic lifestyle shared by all Bathyarchaeota subgroups (Zhou et al., 2018; Feng et al., 2019), the Bathyarchaeota in these sediments could use organic substrates to grow. This was supported by the significantly positive correlation between Bathyarchaeota subgroup 6 abundance and TOC content (Supplementary Table 2). The metabolites produced by Bathyarchaeota might be used by other methanogens, such as members from Methanofastidiosales and Crenarchaeote enrichment culture clone_61-15f detected in the same layer sediments, to produce methane (Supplementary Figure 3; Pavlova et al., 2014; Evans et al., 2019). Furthermore, Bathyarchaeota itself in the bottom clay layers might also have a potential of methanogenesis, which was supported by methane metabolism found in Bathyarchaeota genomes (BA1 and BA2) recovered from a deep aquifer (Evans et al., 2015; Lloyd, 2015). Elusimicrobia, another microbial population appearing in bottom samples A5 and A6 (Figure 4), might contribute to various substrates for methanogens by sugar fermentation to acetate, malate and butyrate, suggested by recent reconstructed Elusimicrobia genomes analysis from groundwater and other natural environments (Meheust et al., 2020). Beyond methanogenesis, these fermentation products could also to be used by the members in class Dehalococcoidia residing in bottom samples A5 and A6 (Figure 4B) to respire the organohalide, a widespread recalcitrant pollutant in groundwater systems (Yang et al., 2020).

### Relationship Between Microbial Community Structure and Sediment Geochemistry

Canonical correspondence analysis was used to determine the most significant geochemical variables to shape the microbial community structure of samples. On the basis of significance (*p* < 0.05) calculated from individual CCA results and VIFs calculated during CCA (Van Nostrand et al., 2009), TOC, HCl-extractable Fe^2+^ and sulfate were chosen from all geochemical variables to perform CCA. The specified CCA model was significant (*p* = 0.014) and described 79.2% of the total variation (the first axis explained 47.3% and the second axis explained 14.3%) (Figure 5A). The microbial community structure in sediments from A1–A2, A3–A4, and A5–A6 appeared to be strongly affected by the concentrations of sulfate, HCl-extractable Fe^2+^ and TOC, respectively, as indicated by their proximity to those arrows in Figure 5A. To further assess the contribution of above three variables to microbial community structure, variation partitioning analysis was performed (Lu et al., 2012a). Three variables explained a large portion of the variation observed, leaving 24.1% of the variation unexplained by these factors. HCl-extractable Fe^2+^ alone accounted for 22.1% (*p* = 0.019), 23.5% of the variation was attributed to sulfate (*p* = 0.026) and TOC explained the largest amount of variation, 25.1% (*p* = 0.013) (Figure 5B). Interactions between and among variables only explained <3.5% of variation. These results suggest that sulfate, HCl-extractable Fe^2+^ and TOC significantly influence the microbial community structures of subsurface clay layers in JHP, which was also reflected by functional potentials of microbial populations.

## Conclusion

Microbial community composition residing in different subsurface clay layers of JHP generally consisted of 67.5% bacterial phyla, 29.5% archaeal phyla, and 3% unclassified phyla. Geochemical and microbial characterizations suggest that the top two clay layers are oxic and the bottom four clay layers are anoxic. High abundance of archaea (up to 60%) were observed in three of the anoxic clay layers. The concentrations of sulfate, HCl-extractable Fe^2+^ and total organic carbon significantly shaped the microbial community structure in different clay layers of subsurface. A distinctly vertical stratification of microbial communities in clay sediments was evident along the borehole. In the upper two oxic clay layers, nitrification, nitrite-dependent methane oxidation, and ammonium oxidation may occur. Reduction of nitrate, iron and sulfate were mainly found in the two anoxic clay layers beneath the oxic zone. The bottom two anoxic clay layers were dominated by archaeal anaerobic degradation of TOC and potential methanogenesis. Additionally, large amounts of unclassified sequences suggested that microorganisms with novel lineages might inhabit in the subsurface clay layers of JHP. These results for the first provide comprehensive insights into bacterial and archaeal community structure and functional potentials in shallow subsurface clay layers of JHP, which will help develop the science-based solutions for mitigating the health risks associated with JHP subsurface.

## Data Availability Statement

The datasets presented in this study can be found in online repositories. The names of the repository/repositories and accession number(s) can be found in the article/Supplementary Material.

## Author Contributions

LS conceived and designed the experiments. DS conducted the field sampling and laboratory experiments. DS and ZJ analyzed the data and wrote the draft manuscript. All authors contributed to the revisions of the manuscript and approved the final manuscript.

## Conflict of Interest

The authors declare that the research was conducted in the absence of any commercial or financial relationships that could be construed as a potential conflict of interest.

## Footnotes

1 https://www.arb-silva.de/

2 http://www.r-project.org/

## References

[B1] Anantharaman K. Brown C. T. Hug L. A. Sharon I. Castelle C. J. Probst A. J. (2016). Thousands of microbial genomes shed light on interconnected biogeochemical processes in an aquifer system. *Nat. Commun.* 7:13219. 10.1038/ncomms13219 27774985 PMC5079060

[B2] Bagnoud A. Chourey K. Hettich R. L. de Bruijn I. Andersson A. F. Leupin O. X. (2016a). Reconstructing a hydrogen-driven microbial metabolic network in Opalinus Clay rock. *Nat. Commun.* 7:12770. 10.1038/ncomms12770 27739431 PMC5067608

[B3] Bagnoud A. de Bruijn I. Andersson A. F. Diomidis N. Leupin O. X. Schwyn B. (2016b). A minimalistic microbial food web in an excavated deep subsurface clay rock. *FEMS Microbiol. Ecol.* 92:fiv138. 10.1093/femsec/fiv138 26542073

[B4] Bell E. Lamminmaki T. Alneberg J. Andersson A. F. Qian C. Xiong W. (2020). Active sulfur cycling in the terrestrial deep subsurface. *ISME J.* 14 1260–1272. 10.1038/s41396-020-0602-x 32047278 PMC7174417

[B5] Boivin-Jahns V. Ruimy R. Bianchi A. Daumas S. Christen R. (1996). Bacterial diversity in a deep-subsurface clay environment. *Appl. Environ. Microbiol.* 62 3405–3412. 10.1128/aem.62.9.3405-3412.1996 8795233 PMC168139

[B6] Castelle C. J. Hug L. A. Wrighton K. C. Thomas B. C. Williams K. H. Wu D. (2013). Extraordinary phylogenetic diversity and metabolic versatility in aquifer sediment. *Nat. Commun.* 4:2120. 10.1038/ncomms3120 23979677 PMC3903129

[B7] Castelle C. J. Wrighton K. C. Thomas B. C. Hug L. A. Brown C. T. Wilkins M. J. (2015). Genomic expansion of domain archaea highlights roles for organisms from new phyla in anaerobic carbon cycling. *Curr. Biol.* 25 690–701. 10.1016/j.cub.2015.01.014 25702576

[B8] Chen X. Sun H. Jiang F. Shen Y. Li X. Hu X. (2020). Alteration of the gut microbiota associated with childhood obesity by 16S rRNA gene sequencing. *PeerJ* 8:e8317. 10.7717/peerj.8317 31976177 PMC6968493

[B9] Daims H. Lucker S. Wagner M. (2016). A new perspective on microbes formerly known as nitrite-oxidizing bacteria. *Trends Microbiol.* 24 699–712. 10.1016/j.tim.2016.05.004 27283264 PMC6884419

[B10] Deng Y. Zheng T. Wang Y. Liu L. Jiang H. Ma T. (2018). Effect of microbially mediated iron mineral transformation on temporal variation of arsenic in the Pleistocene aquifers of the central Yangtze River basin. *Sci. Total Environ.* 619-620 1247–1258. 10.1016/j.scitotenv.2017.11.166 29734603

[B11] Dong H. (2012). Clay-microbe interactions and implications for environmental mitigation. *Elements* 8 95–100. 10.2113/gselements.8.2.113 28159795

[B12] Dong H. Jaisi D. P. Kim J. Zhang G. (2009). Microbe-clay mineral interactions. *Am. Mineral.* 94 1505–1519. 10.2138/am.2009.3246

[B13] Du Y. Ma T. Deng Y. Shen S. Lu Z. (2017). Sources and fate of high levels of ammonium in surface water and shallow groundwater of the Jianghan Plain, central China. *Environ. Sci. Process. Impacts* 19 161–172. 10.1039/c6em00531d 28203672

[B14] Edgar R. C. (2013). UPARSE: highly accurate OTU sequences from microbial amplicon reads. *Nat. Methods* 10 996–998. 10.1038/nmeth.2604 23955772

[B15] Erban L. E. Gorelick S. M. Zebker H. A. Fendorf S. (2013). Release of arsenic to deep groundwater in the Mekong Delta, Vietnam, linked to pumping-induced land subsidence. *Proc. Natl. Acad. Sci. U.S.A* 110 13751–13756. 10.1073/pnas.1300503110 23918360 PMC3752228

[B16] Evans P. N. Boyd J. A. Leu A. O. Woodcroft B. J. Parks D. H. Hugenholtz P. (2019). An evolving view of methane metabolism in the Archaea. *Nat. Rev. Microbiol.* 17 219–232. 10.1038/s41579-018-0136-7 30664670

[B17] Evans P. N. Parks D. H. Chadwick G. L. Robbins S. J. Orphan V. J. Golding S. D. (2015). Methane metabolism in the archaeal phylum Bathyarchaeota revealed by genome-centric metagenomics. *Science* 350 434–438. 10.1126/science.aac7745 26494757

[B18] Fendorf S. Michael H. A. van Geen A. (2010). Spatial and temporal variations of groundwater arsenic in South and Southeast Asia. *Science* 328 1123–1127. 10.1126/science.1172974 20508123

[B19] Feng X. Wang Y. Zubin R. Wang F. (2019). Core metabolic features and hot origin of Bathyarchaeota. *Engineering* 5 498–504. 10.1016/j.eng.2019.01.011

[B20] Fredrickson J. K. Balkwill D. L. (2006). Geomicrobial processes and biodiversity in the deep terrstrial subsurface. *Geomicrobiol. J.* 23 345–356. 10.1080/01490450600875571

[B21] Gan Y. Wang Y. Duan Y. Deng Y. Guo X. Ding X. (2014). Hydrogeochemistry and arsenic contamination of groundwater in the Jianghan Plain, central China. *J. Geochem. Explor.* 138 81–93. 10.1016/j.gexplo.2013.12.013

[B22] Gan Y. Zhao K. Deng Y. Liang X. Ma T. Wang Y. (2018). Groundwater flow and hydrogeochemical evolution in the Jianghan Plain, central China. *Hydrogeol. J.* 26 1609–1623. 10.1007/s10040-018-1778-2

[B23] He Q. Sanford R. A. (2003). Characterization of Fe(III) reduction by chlororespiring Anaeromyxobacter dehalogenans. *Appl. Environ. Microbiol.* 69 2712–2718. 10.1128/aem.69.5.2712-2718.2003 12732541 PMC154556

[B24] Herber J. Klotz F. Frommeyer B. Weis S. Straile D. Kolar A. (2020). A single Thaumarchaeon drives nitrification in deep oligotrophic lake constance. *Environ. Microbiol.* 22 212–228. 10.1111/1462-2920.14840 31657089

[B25] Hesse R. Schacht U. (2011). “Early diagenesis of deep-sea sediments,” in *Deep-Sea Sediments*, eds HüNeke H. Mulder T. (Amsterdam: Elsevier), 557–713. 10.1016/b978-0-444-53000-4.00009-3

[B26] Hug L. A. Castelle C. J. Wrighton K. C. Thomas B. C. Banfield J. F. (2013). Community genomic analyses constrain the distribution of metabolic traits across the Chloroflexi phylum and indicate roles in sediment carbon cycling. *Microbiome* 1 22–22. 10.1186/2049-2618-1-22 24450983 PMC3971608

[B27] Inagaki F. Suzuki M. Takai K. Oida H. Sakamoto T. Aoki K. (2003). Microbial communities associated with geological horizons in coastal subseafloor sediments from the sea of okhotsk. *Appl. Environ. Microbiol.* 69 7224–7235. 10.1128/aem.69.12.7224-7235.2003 14660370 PMC309994

[B28] Islam F. S. Gault A. G. Boothman C. Polya D. A. Charnock J. M. Chatterjee D. (2004). Role of metal-reducing bacteria in arsenic release from Bengal delta sediments. *Nature* 430 68–71. 10.1038/nature02638 15229598

[B29] Jiang Z. Li P. Van Nostrand J. D. Zhang P. Zhou J. Wang Y. (2016). Microbial communities and arsenic biogeochemistry at the outflow of an alkaline sulfide-rich hot spring. *Sci. Rep.* 6:25262. 10.1038/srep25262 27126380 PMC4850476

[B30] Kong Y. (2011). Btrim: a fast, lightweight adapter and quality trimming program for next-generation sequencing technologies. *Genomics* 98 152–153. 10.1016/j.ygeno.2011.05.009 21651976

[B31] Krumholz L. R. McKinley J. P. Ulrich G. A. Sulfita J. W. (1997). Confined subsurface microbial communities in Cretaceous rock. *Nature* 386 64–66. 10.1038/386064a0

[B32] Lin X. Kennedy D. Fredrickson J. Bjornstad B. Konopka A. (2012a). Vertical stratification of subsurface microbial community composition across geological formations at the Hanford Site. *Environ. Microbiol.* 14 414–425. 10.1111/j.1462-2920.2011.02659.x 22122741

[B33] Lin X. Kennedy D. Peacock A. McKinley J. Resch C. T. Fredrickson J. (2012b). Distribution of microbial biomass and potential for anaerobic respiration in Hanford Site 300 Area subsurface sediment. *Appl. Environ. Microbiol.* 78 759–767. 10.1128/AEM.07404-11 22138990 PMC3264105

[B34] Liu H. Liang H. Liang Y. Zhang D. Wang C. Cai H. (2010). Distribution of phthalate esters in alluvial sediment: a case study at JiangHan Plain, Central China. *Chemosphere* 78 382–388. 10.1016/j.chemosphere.2009.11.009 20006896

[B35] Liu X. Li M. Castelle C. J. Probst A. J. Zhou Z. Pan J. (2018). Insights into the ecology, evolution, and metabolism of the widespread Woesearchaeotal lineages. *Microbiome* 6:102. 10.1186/s40168-018-0488-2 29884244 PMC5994134

[B36] Lloyd K. (2015). Beyond known methanogens. *Science* 350 384–384. 10.1126/science.aad4066 26494746

[B37] Lu X. Wang N. Wang H. Deng Y. Ma T. Wu M. (2017). Molecular characterization of the total bacteria and dissimilatory arsenate-Reducing bacteria in core sediments of the Jianghan Plain, Central China. *Geomicrobiol. J.* 34 467–479. 10.1080/01490451.2016.1222468

[B38] Lu Z. Deng Y. Van Nostrand J. D. He Z. Voordeckers J. Zhou A. (2012a). Microbial gene functions enriched in the deepwater horizon deep-sea oil plume. *ISME J.* 6 451–460. 10.1038/ismej.2011.91 21814288 PMC3260509

[B39] Lu Z. He Z. Parisi V. A. Kang S. Deng Y. Van Nostrand J. D. (2012b). GeoChip-based analysis of microbial functional gene diversity in a landfill leachate-contaminated aquifer. *Environ. Sci. Technol.* 46 5824–5833. 10.1021/es300478j 22533634

[B40] Magoc T. Salzberg S. L. (2011). FLASH: fast length adjustment of short reads to improve genome assemblies. *Bioinformatics* 27 2957–2963. 10.1093/bioinformatics/btr507 21903629 PMC3198573

[B41] McMahon P. B. Chapelle F. H. (1991). Microbial productioin of organic acids in aquitard sediments and its role in aquifer geochemistry. *Nature* 349 233–235. 10.1038/349233a0

[B42] McMahon P. B. Chapelle F. H. Falls W. F. Bradley P. M. (1992). Role of microbial processes in linking sandstone diagenesis with organic-rich clays. *J. Sediment. Petrol.* 62 1–10. 10.1306/d4267870-2b26-11d7-8648000102c1865d

[B43] Meheust R. Castelle C. J. Matheus Carnevali P. B. Farag I. F. He C. Chen L. X. (2020). Groundwater *Elusimicrobia* are metabolically diverse compared to gut microbiome *Elusimicrobia* and some have a novel nitrogenase paralog. *ISME J.* [Epub ahead of print]. 10.1038/s41396-020-0716-1 32681159 PMC7785019

[B44] Mihajlov I. Mozumder M. R. H. Bostick B. C. Stute M. Mailloux B. J. Knappett P. S. K. (2020). Arsenic contamination of Bangladesh aquifers exacerbated by clay layers. *Nat. Commun.* 11:2244. 10.1038/s41467-020-16104-z 32382006 PMC7205959

[B45] Muyzer G. Stams A. J. (2008). The ecology and biotechnology of sulphate-reducing bacteria. *Nat. Rev. Microbiol.* 6 441–454. 10.1038/nrmicro1892 18461075

[B46] Pavlova O. N. Bukin S. V. Lomakina A. V. Kalmychkov G. V. Ivanov V. G. Morozov I. V. (2014). Production of gaseous hydrocarbons by microbial communities of Lake Baikal bottom sediments. *Microbiology* 83 798–804. 10.1134/s0026261714060137 25941719

[B47] Shi L. Dong H. Reguera G. Beyenal H. Lu A. Liu J. (2016). Extracellular electron transfer mechanisms between microorganisms and minerals. *Nat. Rev. Microbiol.* 14 651–662. 10.1038/nrmicro.2016.93 27573579

[B48] Svensson S. L. Behroozian S. Xu W. Surette M. G. Li L. Davies J. (2017). Kisameet glacial clay: an unexpected source of bacterial diversity. *mBio* 8:e00590-17. 10.1128/mBio.00590-17 28536287 PMC5442455

[B49] Tong L. Huang S. Wang Y. Liu H. Li M. (2014). Occurrence of antibiotics in the aquatic environment of Jianghan Plain, central China. *Sci. Total Environ.* 497-498 180–187. 10.1016/j.scitotenv.2014.07.068 25128888

[B50] Ulrich G. A. Martino D. Burger K. Routh J. Grossman E. L. Ammerman J. W. (1998). Sulfur cycling in the terrestrial subsurface: commensal interactions, spatial scales, and microbial heterogeneity. *Microb. Ecol.* 36 141–151. 10.1007/s002489900101 9688776

[B51] Van Nostrand J. D. Wu W. M. Wu L. Deng Y. Carley J. Carroll S. (2009). GeoChip-based analysis of functional microbial communities during the reoxidation of a bioreduced uranium-contaminated aquifer. *Environ. Microbiol.* 11 2611–2626. 10.1111/j.1462-2920.2009.01986.x 19624708

[B52] Wang Y. Li P. Jiang Z. Sinkkonen A. Wang S. Tu J. (2016). Microbial community of high arsenic groundwater in agricultural irrigation area of Hetao Plain, Inner Mongolia. *Front. Microbiol.* 7:1917. 10.3389/fmicb.2016.01917 27999565 PMC5138239

[B53] Welte C. U. Rasigraf O. Vaksmaa A. Versantvoort W. Arshad A. Op (2016). Nitrate- and nitrite-dependent anaerobic oxidation of methane. *Environ. Microbiol. Rep.* 8 941–955. 10.1111/1758-2229.12487 27753265

[B54] Wong D. Suflita J. M. McKinley J. P. Krumholz L. R. (2004). Impact of clay minerals on sulfate-reducing activity in aquifers. *Microb. Ecol.* 47 80–86. 10.1007/s00248-003-1021-z 15259272

[B55] Wu G. Yang J. Jiang H. Deng Y. Lear G. (2019). Distribution of potentially pathogenic bacteria in the groundwater of the Jianghan Plain, central China. *Int. Biodeterior. Biodegrad.* 143:104711 10.1016/j.ibiod.2019.05.028

[B56] Xiao C. Ma T. Du Y. Liu Y. Liu R. Zhang D. (2020). Impact process of the aquitard to regional arsenic accumulation of the underlying aquifer in Central Yangtze River Basin. *Environ. Geochem. Health* [Epub ahead of print]. 10.1007/s10653-020-00541-2 32839956

[B57] Yang Y. Sanford R. Yan J. Chen G. Capiro N. L. Li X. (2020). Roles of organohalide-respiring *Dehalococcoidia* in carbon cycling. *mSystems* 5:e00757-19. 10.1128/mSystems.00757-19 PMC7289593 32518199

[B58] Zheng T. Deng Y. Wang Y. Jiang H. O’Loughlin E. J. Flynn T. M. (2019). Seasonal microbial variation accounts for arsenic dynamics in shallow alluvial aquifer systems. *J. Hazard. Mater.* 367 109–119. 10.1016/j.jhazmat.2018.12.087 30594709

[B59] Zhou Z. Pan J. Wang F. Gu J. D. Li M. (2018). Bathyarchaeota: globally distributed metabolic generalists in anoxic environments. *FEMS Microbiol. Rev.* 42 639–655. 10.1093/femsre/fuy023 29790926

