# Supplementary material for: Bacterial and Archaeal Diversity and Abundance in Shallow Subsurface Clay Sediments at Jianghan Plain, China
Source: Front Microbiol. 2020 Oct 22;11:572560. doi: 10.3389/fmicb.2020.572560 (PMC7642157; doi:10.3389/fmicb.2020.572560)
Supplement: Supplementary file 1 [file Data_Sheet_1.docx]

Supplementary Material

# Supplementary Tables

**Supplementary Table 1.** Alpha diversity indices at a 97% similarity level of 16S rRNA gene fragments by re-sampling 42887 sequences in each sample.

| Samples | Observed OTUs | Chao1 | Coverage ^a^ of observed OTUs (%) | Shannon' diversity | Equitability |
| --- | --- | --- | --- | --- | --- |
| A1 | 2829 | 3255 | 86.90 | 6.14 | 0.77 |
| A2 | 3848 | 4250 | 90.54 | 6.81 | 0.83 |
| A3 | 2559 | 3046 | 84.02 | 5.70 | 0.73 |
| A4 | 2268 | 2492 | 91.02 | 5.95 | 0.77 |
| A5 | 1990 | 2521 | 78.95 | 5.36 | 0.71 |
| A6 | 2138 | 2589 | 82.57 | 5.65 | 0.74 |
| ^a^ Coverage is the ratio of the observed OTUs to Chao1 | | | | | |

**Supplementary Table 2.** Correlation analysis (r values) between microbial community composition abundance and geochemical variables. Only microbial community composition in Figure 4 and Figure S2 were listed below.

| Microbial community compositions | Depth | pH | TOC | NO_3_^-^ | SO_4_^2-^ | HCl-extractable Fe^2+^ | Fe_Tot_ | Mn_Tot_ | As_Tot_ |
| --- | --- | --- | --- | --- | --- | --- | --- | --- | --- |
| **Phylum** |  |  |  |  |  |  |  |  |  |
| Chloroflexi | +0.038 | -0.719 | +0.660 | -0.518 | -0.651 | +0.865* | +0.746 | +0.817* | +0.321 |
| Proteobacteria | -0.247 | -0.089 | +0.009 | -0.333 | -0.215 | +0.597 | +0.753 | +0.557 | +0.844* |
| Acidobacteria | -0.604 | +0.777 | -0.416 | +0.620 | +0.955** | -0.844* | -0.616 | -0.152 | +0.220 |
| Planctomycetes | +0.724 | -0.120 | +0.870* | +0.383 | -0.167 | +0.263 | +0.183 | +0.384 | -0.362 |
| Actinobacteria | -0.236 | -0.383 | -0.340 | -0.382 | -0.335 | -0.189 | -0.542 | -0.639 | -0.737 |
| Patescibacteria | -0.643 | +0.526 | -0.316 | +0.450 | +0.822* | -0.786 | -0.641 | -0.088 | +0.115 |
| Elusimicrobia | +0.880* | -0.145 | +0.751 | +0.279 | -0.226 | +0.291 | +0.254 | +0.265 | -0.332 |
| NC10 | -0.591 | +0.710 | -0.570 | +0.462 | +0.890* | -0.830* | -0.588 | -0.261 | +0.254 |
| Firmicutes | -0.460 | +0.119 | -0.488 | -0.246 | -0.084 | +0.136 | +0.161 | -0.131 | +0.364 |
| Bathyarchaeota | +0.852* | -0.532 | +0.567 | -0.166 | -0.638 | +0.440 | +0.214 | -0.020 | -0.610 |
| Euryarchaeota | -0.712 | +0.640 | -0.689 | +0.213 | +0.700 | -0.523 | -0.238 | -0.133 | +0.604 |
| Thaumarchaeota | -0.584 | +0.874* | -0.539 | +0.634 | +0.954** | -0.840* | -0.587 | -0.256 | +0.256 |
| Woesearchaeota | -0.632 | +0.863* | -0.595 | +0.584 | +0.878* | -0.783 | -0.562 | -0.307 | +0.247 |
| **Class** |  |  |  |  |  |  |  |  |  |
| Anaerolineae | -0.370 | -0.499 | -0.039 | -0.717 | -0.503 | +0.674 | +0.660 | +0.453 | +0.615 |
| Dehalococcoidia | +0.587 | -0.308 | +0.905* | +0.236 | -0.242 | +0.278 | +0.128 | +0.435 | -0.420 |
| Deltaproteobacteria | -0.092 | -0.462 | +0.224 | -0.582 | -0.528 | +0.833* | +0.878* | +0.660 | +0.700 |
| Aminicenantia | +0.712 | -0.505 | +0.882* | -0.085 | -0.618 | +0.721 | +0.570 | +0.522 | -0.198 |
| Phycisphaerae | +0.682 | -0.379 | +0.931** | +0.138 | -0.380 | +0.448 | +0.303 | +0.481 | -0.347 |
| Microgenomatia | -0.755 | +0.347 | -0.560 | +0.163 | +0.643 | -0.773 | -0.713 | -0.322 | +0.004 |
| Elusimicrobia 4-29 | +0.879* | -0.148 | +0.759 | +0.279 | -0.233 | +0.300 | +0.259 | +0.271 | -0.334 |
| Methylomirabilales | -0.577 | +0.700 | -0.604 | +0.448 | +0.878* | -0.858* | -0.631 | -0.326 | +0.195 |
| Bathyarchaeota  Subgroup 6 | +0.764 | -0.336 | +0.892* | +0.160 | -0.375 | +0.435 | +0.310 | +0.416 | -0.358 |
| Bathyarchaeota  Subgroup 12 | +0.329 | -0.542 | -0.157 | -0.471 | -0.587 | +0.058 | -0.270 | -0.638 | -0.808 |
| Bathyarchaeota  Subgroup 11 | -0.253 | -0.469 | -0.341 | -0.752 | -0.614 | +0.502 | +0.365 | -0.105 | +0.175 |
| Thermoplasmata | -0.753 | +0.711 | -0.668 | +0.360 | +0.818* | -0.710 | -0.469 | -0.223 | +0.410 |
| Nitrososphaeria | -0.589 | +0.873* | -0.532 | +0.636 | +0.954** | -0.834* | -0.581 | -0.245 | +0.263 |
| **Order/Family/Genus** |  |  |  |  |  |  |  |  |  |
| Methanosarcinales | -0.242 | -0.648 | -0.342 | -0.915* | -0.699 | +0.517 | +0.349 | -0.116 | +0.124 |
| Methanocellales | -0.211 | -0.371 | +0.062 | -0.578 | -0.444 | +0.738 | +0.809 | +0.568 | +0.750 |
| Methanobacteriales | -0.185 | -0.534 | -0.113 | -0.789 | -0.630 | +0.732 | +0.702 | +0.291 | +0.524 |
| Methanofastidiosales | +0.900* | -0.229 | +0.765 | +0.117 | -0.371 | +0.542 | +0.551 | +0.424 | -0.081 |
| Crenarchaeote  enrichment culture  clone 61-15f | +0.557 | -0.266 | +0.917** | +0.286 | -0.215 | +0.265 | +0.111 | +0.445 | -0.423 |
| Chloroflexi bacterium  RBG_16_58_14 | -0.272 | -0.293 | -0.004 | -0.528 | -0.394 | +0.679 | +0.748 | +0.507 | +0.735 |
| *Anaeromyxobacter* | -0.297 | -0.274 | -0.010 | -0.518 | -0.346 | +0.655 | +0.752 | +0.540 | +0.790 |
| *‘Candidatus*  *Methanoperedens'* | -0.238 | -0.656 | -0.336 | -0.918** | -0.704 | +0.520 | +0.349 | -0.114 | +0.118 |
| Nitrospirales | -0.595 | +0.861* | -0.521 | +0.631 | +0.957** | -0.838* | -0.586 | -0.232 | +0.263 |
| Nitrospinae | -0.649 | +0.778 | -0.611 | +0.502 | +0.909* | -0.838* | -0.599 | -0.285 | +0.267 |
| *Nitrosomonas* | -0.594 | +0.745 | -0.623 | +0.500 | +0.696 | -0.752 | -0.663 | -0.492 | -0.028 |
| Nitrososphaerales | -0.528 | +0.905* | -0.501 | +0.678 | +0.861* | -0.746 | -0.537 | -0.286 | +0.192 |
| Nitrosopumilales | -0.588 | +0.850* | -0.523 | +0.617 | +0.957** | -0.836* | -0.577 | -0.226 | +0.276 |

+ and -: positive and negative relationship, respectively. *: p < 0.05; **: p < 0.01

**Supplementary Table 3.** Significance tests of microbial community structure difference among groups with three complementary statistical approaches.

| Data sets | adonis^a^ | | ANOSIM^b^ | | MRPP^c^ | |
| --- | --- | --- | --- | --- | --- | --- |
|  | *F* | *P* | *R* | *P* | δ | *P* |
| Group 1 (samples A1 and A2)  Group 2 (samples A3 and A4)  Group 3 (samples A5 and A6) | 3.1985 | 0.022 | 1 | 0.066 | 0.8054 | 0.066 |

All tests are non-parametric multivariate analyses based on Bray-Curtis dissimilarities among samples. a adonis: Permutational multivariate analysis of variance using distance matrices. Significance tests were carried out using F-tests based on sequential sums of squares from permutations of the raw data. b ASNOSIM: Analysis of similarities which provides a way to test statistically whether there is a significant difference between two or more groups of sampling units. Statistic R is based on the difference of mean ranks between groups and within groups. The significance of observed R is assessed by permuting the grouping vector to obtain the empirical distribution of R under the null model. c MRPP: Multi-response permutation procedure. Statistic delta is the overall weighted mean of within-group means of the pairwise dissimilarities among sampling units. The significance test is the fraction of permuted delta that is less than the observed delta.

# Supplementary Figures


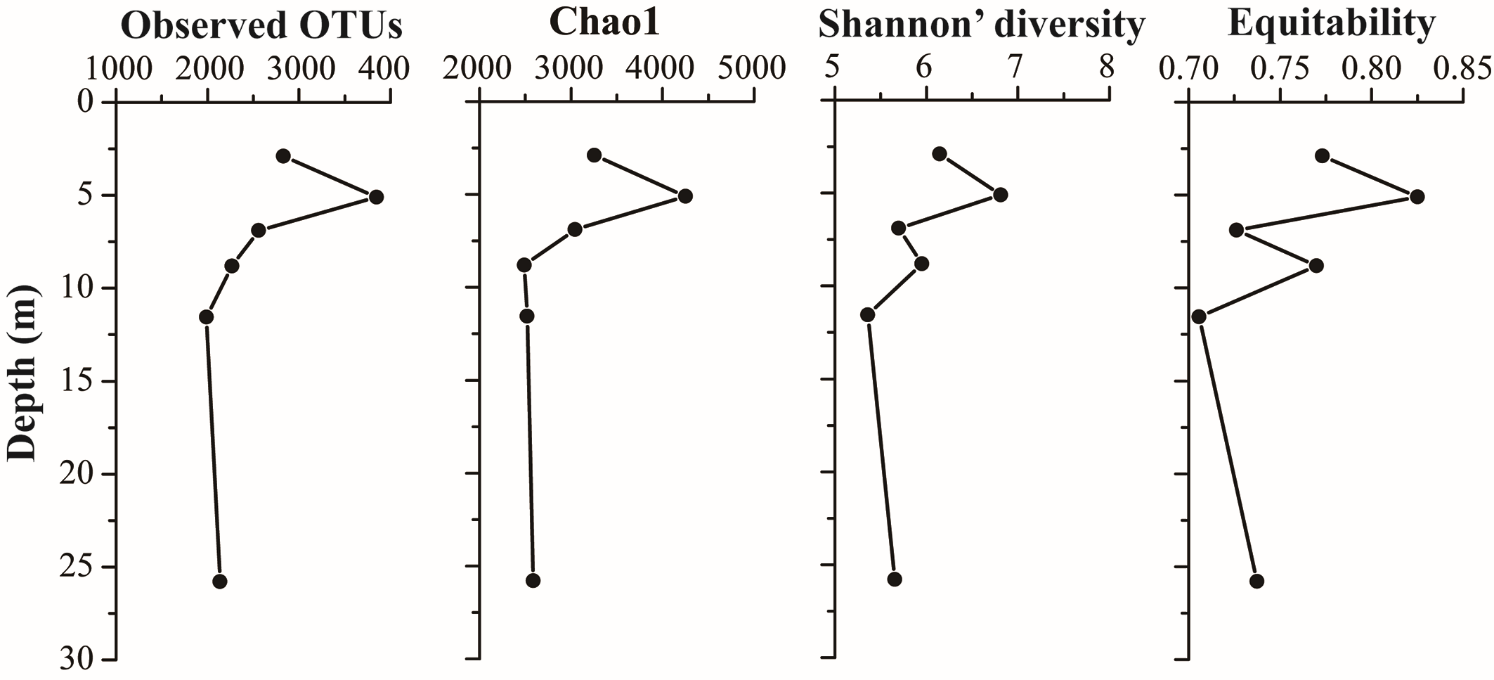


**Supplementary Figure 1.** Vertical distribution of alpha diversity indices of microbial community composition in samples.


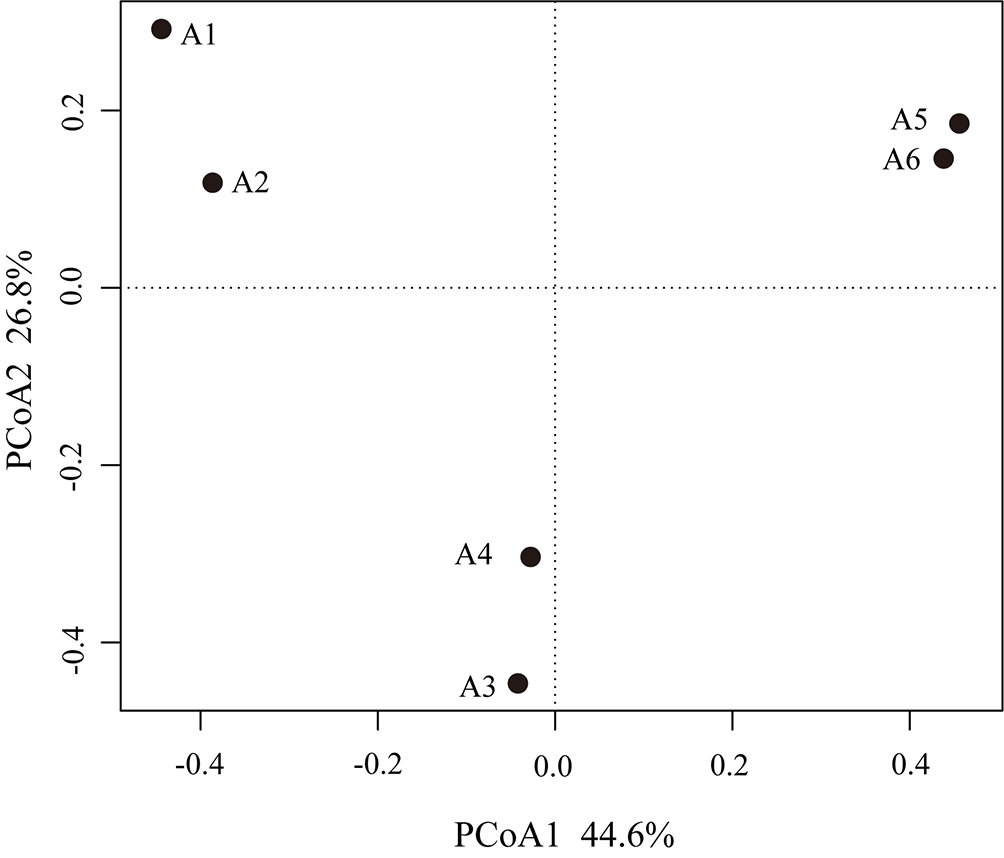


**Supplementary Figure 2.** Principal coordinates analysis (PCoA) of microbial community composition residing in subsurface clay sediments in JPH.


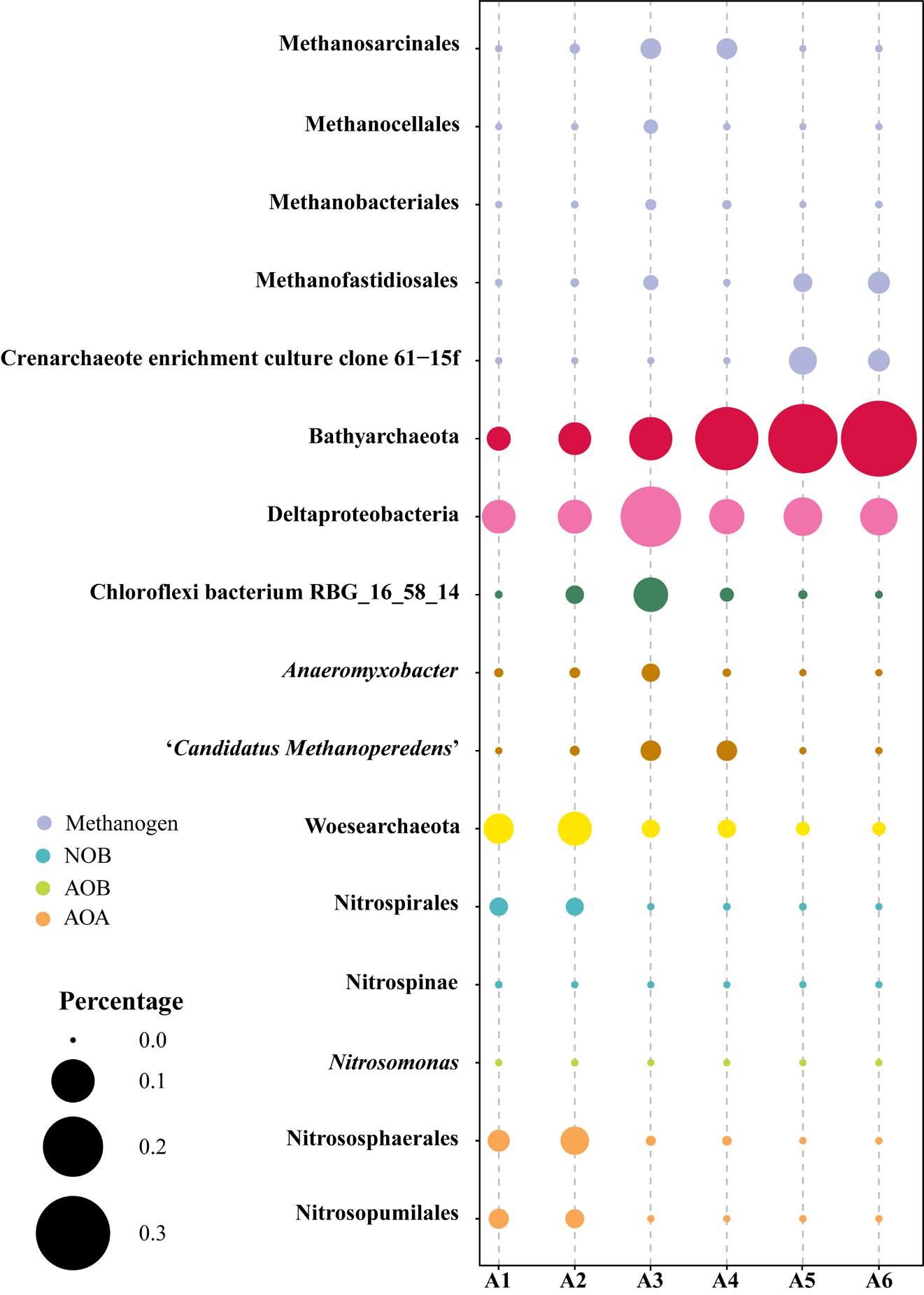


**Supplementary Figure 3.** Vertical distribution of microbial community composition associated with nitrogen, iron, sulfate and carbon metabolisms. Only representative functional populations with distinct distribution difference alone the borehole and relatively higher abundance are displayed.
